# The microbiota is dispensable for the early stages of peripheral regulatory T cell induction within mesenteric lymph nodes

**DOI:** 10.1038/s41423-021-00647-2

**Published:** 2021-03-24

**Authors:** Carolin Wiechers, Mangge Zou, Eric Galvez, Michael Beckstette, Maria Ebel, Till Strowig, Jochen Huehn, Joern Pezoldt

**Affiliations:** 1 Department Experimental Immunology, Helmholtz Centre for Infection Research, Braunschweig, Germany; 2 Department Microbial Immune Regulation, Helmholtz Centre for Infection Research, Braunschweig, Germany; 3 Department of Computational Biology for Individualised Medicine, Centre for Individualised Infection Medicine, Helmholtz Centre for Infection Research and Hannover Medical School, Hannover, Germany; 4 Cluster of Excellence RESIST (EXC 2155), Hannover Medical School, Hannover, Germany; 5 Laboratory of Systems Biology and Genetics, École Polytechnique Fédérale de Lausanne, Lausanne, Switzerland

**Keywords:** Peripheral regulatory T cells, Microbiota, Tolerance, Mucosal immunology, Peripheral tolerance

## Abstract

Intestinal Foxp3^+^ regulatory T cell (Treg) subsets are crucial players in tolerance to microbiota-derived and food-borne antigens, and compelling evidence suggests that the intestinal microbiota modulates their generation, functional specialization, and maintenance. Selected bacterial species and microbiota-derived metabolites, such as short-chain fatty acids (SCFAs), have been reported to promote Treg homeostasis in the intestinal lamina propria. Furthermore, gut-draining mesenteric lymph nodes (mLNs) are particularly efficient sites for the generation of peripherally induced Tregs (pTregs). Despite this knowledge, the direct role of the microbiota and their metabolites in the early stages of pTreg induction within mLNs is not fully elucidated. Here, using an adoptive transfer-based pTreg induction system, we demonstrate that neither transfer of a dysbiotic microbiota nor dietary SCFA supplementation modulated the pTreg induction capacity of mLNs. Even mice housed under germ-free (GF) conditions displayed equivalent pTreg induction within mLNs. Further molecular characterization of these *de novo* induced pTregs from mLNs by dissection of their transcriptomes and accessible chromatin regions revealed that the microbiota indeed has a limited impact and does not contribute to the initialization of the Treg-specific epigenetic landscape. Overall, our data suggest that the microbiota is dispensable for the early stages of pTreg induction within mLNs.

## Introduction

Foxp3^+^ regulatory T cells (Tregs) are key mediators of immune tolerance.^1^ While the vast majority of Tregs are thymus derived (tTregs), preferentially recognize self-antigens and prevent autoimmunity,^2,3^ a substantial proportion of Tregs develop in the periphery via conversion of naive CD4^+^ T cells into Foxp3^+^ Tregs, termed peripherally induced Tregs (pTregs).^4^ Numerous studies have reported the preferential occurrence of pTregs at intestinal sites and their associated secondary lymphoid organs, reflecting the necessity of constant fine-tuning to sustain immune tolerance toward the high abundance of harmless commensal and food-borne antigens.^5–9^

Others and us have shown that the initial stages of *de novo* pTreg induction most efficiently take place in gut-draining lymph nodes (LNs), including the mesenteric and celiac lymph nodes (mLNs and celLNs, respectively), particularly when compared to skin-draining peripheral LNs (pLNs).^5,10–13^ Efficient pTreg induction in gut-draining LNs relies on dendritic cells (DCs) loaded with gut-derived antigens, which either directly migrate from the lamina propria to the gut-draining LNs or sample the LN conduit system to present processed antigen to naive T cells.^5,13,14^ It is widely accepted that the tolerogenic gut-specific microenvironment comprising elevated levels of retinoic acid and TGFβ, together with an overrepresentation of tolerogenic CD103^+^ DCs, enables efficient initial pTreg induction in gut-draining LNs.^5,15,16^ The primed T cells then proliferate and attain gut-tropic features, thus acquiring the ability to subsequently home to the intestinal lamina propria.^17–19^ Here, they reencounter their cognate antigens and undergo further differentiation and expansion in a second step of pTreg induction that is propelled by intestinal macrophages.^20,21^

Several bacterial species and metabolites directly foster Treg homeostasis in the colonic lamina propria and associated lymphoid structures,^22^ underscored by the immunological deficits of germ-free (GF) mice.^23^ Remarkably, colonization of adult GF animals with the altered Schaedler flora,^24^ *Clostridium* species,^25^ or *Bacteroides fragilis*^26^ was already sufficient to increase either the proportion of gut-resident Tregs or their tolerogenic functional capacity.^22^ Importantly, only a few select bacterial metabolites have been identified to promote the stability and expansion of pTregs, including bile acid metabolites^27–29^ and short-chain fatty acids (SCFAs), which are produced by multiple symbionts, including *Bifidobacteria* and *Clostridium butyricum*.^4,22^ Dietary supplementation with SCFAs alone increased Treg numbers in antibiotic-treated mice^30^ and could even ameliorate colitis symptoms.^31,32^ On the other hand, several studies have shown that a high abundance of TM7*, Prevotellaceae* or *Helicobacter hepaticus* results in a dysbiotic microbiota composition and drives the development of inflammatory bowel disease.^33–37^ The fact that inflammatory bowel diseases are also exacerbated in the presence of a dysfunctional Treg compartment thus highlights the functional link between Tregs and microbiota homeostasis and their importance for lamina propria tissue integrity.^38^ Nevertheless, despite a growing body of evidence regarding the microbial modulation of the Treg population residing inside the intestinal lamina propria, the direct impact of the microbiota on *de novo* Treg induction at its earliest stage, the initial priming within gut-draining LNs, has yet to be elucidated. We thus aimed to discover how selected microbial metabolites, a dysbiotic microbiota composition or the complete absence of a microbiota affect the extent of pTreg induction and their phenotype in mLNs.

Here, we report that the initial priming of *de novo* induced Tregs is a robust intrinsic feature of mLNs and independent of the microbiota. Importantly, autonomy from the microbiota is not only limited to the frequency of *de novo* induced Tregs but also extends to their transcriptional and epigenetic landscape, indicating that the mLN microenvironment drives early stages of pTreg differentiation regardless of the presence of a microbiota to set the stage for targeted modulation of pTregs in the intestine.

## Results

### SCFA supplementation does not affect the pTreg induction capacity of mLNs

**Fig. 1:**
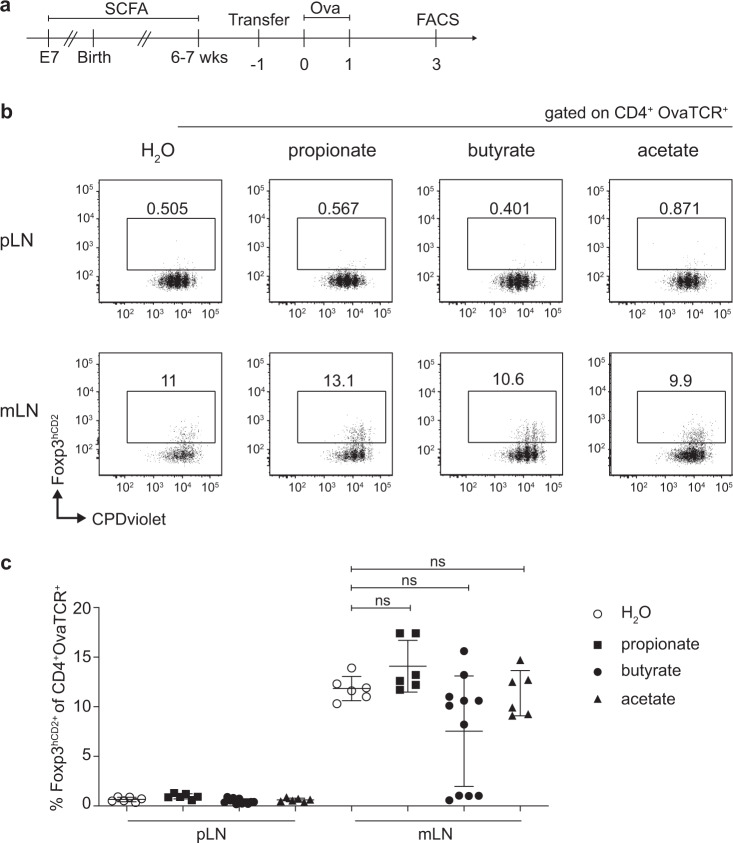
SCFA supplementation does not increase *de novo* pTreg generation in mLNs. **a** Either propionate, butyrate or acetate supplementation (100 mM) was initiated via the mother’s drinking water at E7 during gestation. Respective SCFA supplementation continued for the offspring until six to seven weeks of age, when treated mice and age-matched controls received CPDviolet-labeled naive CD4^+^ T cells from Foxp3^hCD2^xRag2^*−/−*^xDO11.10 mice. One day later, recipients were immunized via repetitive i.v. injection with Ova_323-339_ peptide on two consecutive days and analyzed on day 3 after the first immunization. **b** Exemplary dot plots depict *de novo* pTreg induction in the indicated LNs and treatment conditions gated on adoptively transferred OvaTCR^+^CD4^+^ T cells. **c** The scatterplot summarizes the frequencies of *de novo* induced Foxp3^+^ pTregs among transferred OvaTCR^+^CD4^+^ T cells recovered from the pLNs or mLNs of SCFA-treated or untreated mice. Data pooled from two independent experiments with bars indicating the mean ± SD (*n* = 6–7, per condition). i.v., intravenous; mLNs, mesenteric lymph nodes; Ova, ovalbumin; pLNs, peripheral lymph nodes; pTregs, peripherally induced regulatory T cells; SCFA, short-chain fatty acid; SPF, specific pathogen-free; TCR, T cell receptor

SCFAs are known to promote the stability and expansion of intestinal Tregs, thereby playing an active role in establishing and maintaining a tolerogenic microenvironment within the intestinal immune system. These bacterial metabolites act both locally on intestinal immune cells, e.g., by promoting the induction of colonic Treg differentiation,^30–32,39^ and systemically once they enter the bloodstream via the portal vein.^40,41^ Here, we aimed to elucidate whether SCFAs can also foster the early stages of pTreg induction in mLNs. To this end, SPF-housed mice were treated orally by drinking water with either 100 mM propionate, butyrate, or acetate. SCFA supplementation was initiated on embryonic day 7 (E7) during gestation via the mother’s drinking water and continued until the offspring reached 6 to 7 weeks of age. To test whether supplementation with distinct SCFAs can modulate the pTreg induction capacity of mLNs, we utilized a pTreg induction system based on the adoptive transfer of ovalbumin (Ova)-specific naive Foxp3^−^CD4^+^ T cells from DO11.10 TCR-transgenic mice into recipient mice followed by systemic intravenous (i.v) Ova application and assessment of the frequency of *de novo* induced pTregs at day 3 after antigen application, an early time point at which egress from the LN and subsequent recirculation have not yet occurred (Fig. 1a). Flow cytometric analysis revealed that SCFA treatment did not alter the proliferation of Ova-specific T cells, which was comparable within all analyzed LNs (Fig. 1b). In accordance with our previous observations,^16,42^ pTreg induction was significantly higher in mLNs than in pLNs. However, neither propionate, butyrate nor acetate supplementation resulted in a significant increase in *de novo* induced pTregs in mLNs compared to those in mLNs from untreated controls (Fig. 1b, c). Furthermore, the frequencies of endogenous CD4^+^ T cells and Tregs in pLNs and mLNs were also unaffected upon SCFA supplementation (Supplementary Fig. S1a, b). We also assessed the CD4^+^ T cell population within the colonic lamina propria; however, in contrast to previously published studies,^30,32^ we did not observe an increased frequency or number of Foxp3^+^ Tregs upon dietary SCFA supplementation (Supplementary Fig. 1c–e). However, SCFA supplementation was effective since adoptively transferred Ova-specific T cells showed a weak but significantly increased induction of CCR9 expression in mLNs of acetate-treated mice (Supplementary Fig. S1f). Together, our data indicate that dietary SCFA supplementation does not affect the pTreg induction capacity of mLNs.

### pTreg induction in mLNs is maintained in the presence of a dysbiotic microbiota

**Fig. 2:**
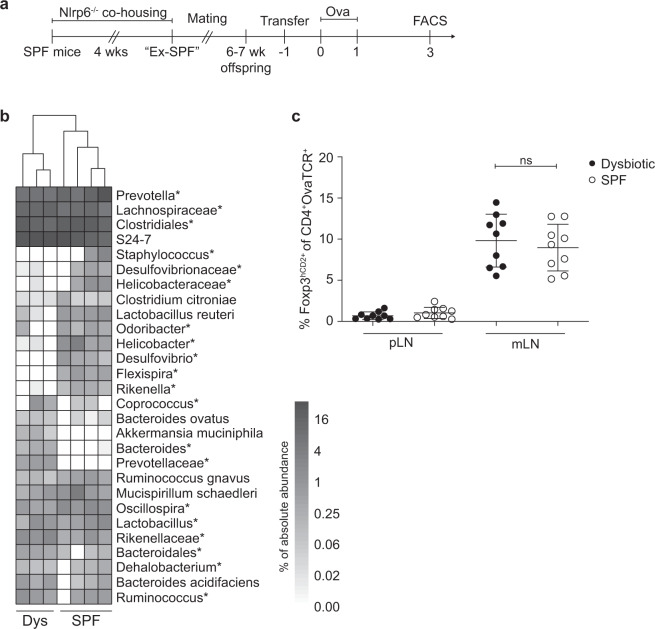
The presence of a dysbiotic colitogenic microbiota does not diminish pTreg induction. **a** Seven- to twelve-week-old female *Nlrp6*^*−/−*^ mice were cohoused with five- to six-week-old female SPF mice (BALB/c) for 4 weeks. After cohousing, the Ex-SPF BALB/c mice were mated to obtain offspring to assess *de novo* Treg induction. When offspring reached 6–7 weeks of age, mice received CPDviolet-labeled naive CD4^+^ T cells from Foxp3^hCD2^xRag2^*−/−*^xDO11.10 mice. One day later, recipients were immunized via repetitive i.v. injection with Ova_323-339_ peptide on two consecutive days and analyzed on day 3 after the first immunization. **b** Fecal samples of adoptive cell transfer recipients were collected for 16S rDNA analysis of the V4 region. The heatmap depicts the absolute abundance frequencies of genera and families/orders (indicated with “*”). **c** Scatterplot displaying the frequencies of *de novo* induced Foxp3^+^ pTregs among transferred OvaTCR^+^CD4^+^ T cells recovered from pLNs and mLNs of SPF control, and dysbiotic mice. Data pooled from two independent experiments; bars indicate the mean ± SD (*n* = 9, per condition). mLNs, mesenteric lymph nodes; Ova, ovalbumin; pLNs, peripheral lymph nodes; pTregs, peripherally induced regulatory T cells; SPF, specific pathogen-free; TCR, T cell receptor

Next, we hypothesized that a disruption of the homeostatic intestinal microbiota via the establishment of pathobionts could deregulate pTreg induction within mLNs. To this end, we employed *Nlrp6*^*−/−*^ mice, which harbor a colitogenic microbiome driven by deficiency of the key inflammasome component Nlrp6. The microbiome of *Nlrp6*^*−/−*^ mice is enriched for *Prevotellaceae*, a state that we here term “dysbiotic” and which enhances susceptibility to mucosal inflammation upon transfer,^35,36^ and can readily be transferred across mice via cohousing. Adult *Nlrp6*^*−/−*^ mice were cohoused with 5- to 6-week-old specific pathogen-free (SPF) mice for a period of 4 weeks, and former SPF mice (Ex-SPF) were mated (Fig. 2a). The successful transfer of the dysbiotic microbiota was verified by 16S rDNA sequencing of fecal samples from their offspring. Key characteristic shifts in the intestinal microbiota composition were observed, including an accumulation of *Prevotellaceae* (Fig. 2b) along with a global depletion of *Proteobacteria* (Supplementary Fig. S2), which was previously reported to be a hallmark of the inflammation-driving microbiota-associated phenotype.^35^ Among endogenous (OvaTCR^-^) CD4^+^ T cells, the frequencies of CD45RB^high^, CD45RB^low^ and CD69^+^ subsets, serving as a proxy for naive, effector/memory and recently activated CD4^+^ T cells, respectively, remained unaltered in pLNs and mLNs (Supplementary Fig. 3a–c), indicating that the CD4^+^ T cell compartment was not affected by the dysbiotic microbiota. However, we observed a tendency for an increased proportion and number of CD103^+^ DCs in mLNs (Supplementary Fig. S4a–c), suggesting elevated DC trafficking from the gut lamina propria. Importantly, when we next assessed pTreg induction in pLNs and mLNs using the aforementioned adoptive transfer-based system, no difference in the frequency of *de novo* induced pTregs could be observed in pLNs and mLNs between dysbiotic and SPF control mice (Fig. 2c). To further dissect to what extent local microbiota changes could affect *de novo* Treg induction, we assessed the pTreg induction potential of LNs draining the small intestine or the cecum/upper colon in SPF mice. However, we could not observe differences between small intestine-draining LNs and cecum-draining LNs (Supplementary Fig. S5). Together, our data suggest that efficient pTreg induction in mLNs is a stable feature along the intestine and remains unaltered even subsequent to transfer of a colitogenic microbiota.

### The microbiota is dispensable for pTreg induction within mLNs

**Fig. 3:**
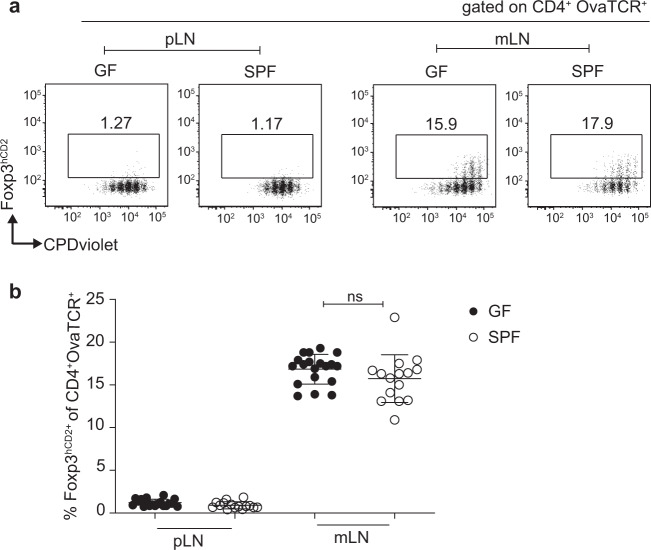
The microbiota is dispensable for pTreg induction in mLNs. Four- to eight-week-old GF and SPF-housed mice received CPDviolet-labeled naive CD4^+^ T cells from Foxp3^hCD2^xRag2^*−/−*^xDO11.10 mice. One day later, recipients were immunized via repetitive i.v. injection with Ova_323-339_ peptide on two consecutive days and analyzed on day 3 after the first immunization. **a** Example dot plots depict *de novo* pTreg induction in the indicated LNs of either GF- or SPF-housed mice gated on adoptively transferred OvaTCR^+^CD4^+^ T cells. **b** Scatterplot displaying the frequencies of *de novo* induced Foxp3^+^ pTregs among transferred OvaTCR^+^CD4^+^ T cells in the indicated LNs of either GF- or SPF-housed mice. Data pooled from two independent experiments, with bars indicating the mean ± SD (*n* = 15-19). GF, germ-free; i.v., intravenous; mLNs, mesenteric lymph nodes; Ova, ovalbumin; pLN, peripheral lymph node; pTregs, peripherally induced regulatory T cells; SPF, specific pathogen-free; TCR, T cell receptor

Since neither dietary SCFA supplementation nor the transfer of a dysbiotic microbiota affected *de novo* Treg induction in mLNs, we next aimed to identify whether the microbiota contributes to pTreg induction within mLNs. To this end, we assessed GF mice, which allowed us to study the impact of the complete absence of a microbiota on the early stages of pTreg induction in mLNs. First, we evaluated the Treg subset composition within the intestinal immune system in GF mice. In line with previously published studies,^24,25,43,44^ we observed a significantly reduced frequency of RORγt^+^ Tregs in both mLNs and particularly within the colonic lamina propria of GF mice when compared to that of SPF mice, which was accompanied by an increase in the frequency of Helios^+^ Tregs (Supplementary Fig. S6a, b). These data demonstrate the relevance of the microbiota for shaping characteristic tissue-resident Treg subsets in the intestine. When we next analyzed pTreg induction in mLNs; surprisingly, we observed a comparable frequency of *de novo* induced pTregs in GF and age-matched SPF-housed control mice (Fig. 3a, b). As expected, pTreg induction was consistently lower within the pLNs of GF mice than in the mLNs, mirroring the phenotype of SPF controls. This striking similarity of pTreg induction in the mLNs of GF and SPF-housed mice suggests that the microbiota is dispensable for the early stages of pTreg induction in mLNs, which is in stark contrast to the impact of the microbiota on Treg subset composition in mucosa-associated tissues, such as the colonic lamina propria.

### The intrinsic properties of mLNs shape transcriptional and epigenetic priming of pTregs irrespective of microbial colonization

Having demonstrated that an equal frequency of pTregs is induced in the mLNs of GF and SPF-housed mice, we next asked if the microbiota might have an impact on the transcriptome and epigenome of the newly generated pTregs in mLNs. To this end, we adoptively transferred Ova-specific naive Foxp3^hCD2−^CD4^+^ T cells from Foxp3^hCD2^xRag2^*−/−*^xDO11.10 mice into GF and SPF-housed recipient mice followed by systemic Ova application. At day 3 after antigen application, *de novo* induced Foxp3^hCD2+^CD4^+^OvaTCR^+^ pTregs were sorted from mLNs of GF (pTreg GF) and SPF-housed mice (pTreg SPF) and utilized for RNA-seq and transposase accessible chromatin (ATAC)-seq analyses. Naive CD4^+^OvaTCR^+^CD62L^+^ T cells from Foxp3^hCD2^xRag2^*−/−*^xDO11.10 mice were taken as input controls.

**Fig. 4:**
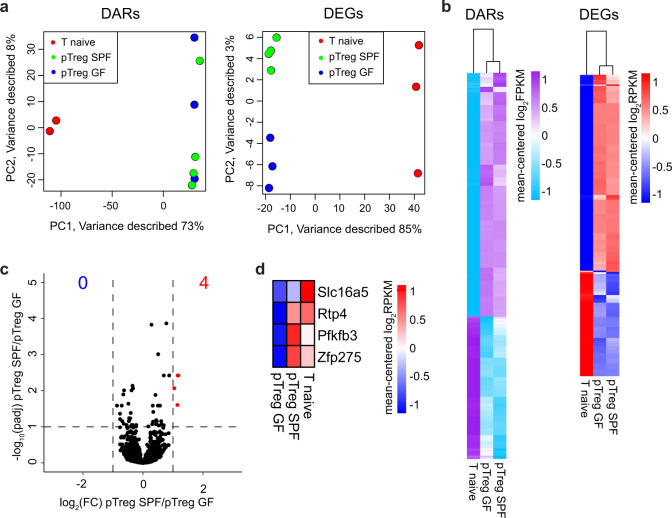
The transcriptome and chromatin accessibility of *de novo* induced pTregs is independent of microbial colonization. *De novo* induced Foxp3^hCD2+^ pTregs among adoptively transferred CD4^+^OvaTCR^+^ T cells were isolated from mLNs of GF and SPF-housed mice after Ova immunization, and CD62L^+^CD4^+^OvaTCR^+^ naive T cells were freshly isolated from Foxp3^hCD2^xRag2^*−/−*^xDO11.10 mice. Subsequently, RNA-seq and ATAC-seq analyses were carried out. **a** PCA based on DARs localized at the TSS identified in cell type-specific (pTregs vs naive T cells) and colonization-dependent (GF vs SPF) pairwise comparisons (log_2_(FC) ≥ 1, *q*-value ≤ 0.05) **(left)**, and PCA based on DEGs identified in cell type-specific (pTregs vs naive T cells) and colonization-dependent (GF vs SPF) pairwise comparisons (log_2_(FC) ≥ 1, *q*-value ≤ 0.05) **(right)**. **b** Hierarchical clustering of 5134 DARs mapped at TSSs (mean-centered log2(FPKM) values, **(left)**) and hierarchical clustering of 837 DEGs (mean-centered log_2_(RPKM) values, **(right)**) are depicted. **c** Volcano plot depicting log_2_(FC) vs. −log_10_(padj) of identified DEGs between SPF and GF pTregs. **d** Heatmap displaying the expression of the four significantly upregulated genes between GF and SPF pTregs. Samples for both ATAC-seq and RNA-seq were pooled from two independent experiments (*n* = 2–4). ATAC, assay for transposase accessible chromatin; DAR, differentially accessible region; DEG, differentially expressed gene; FC, fold change; FPKM, fragments per kilobase of peak per million reads; GF, germ-free; mLNs, mesenteric lymph nodes; padj, FDR-adjusted *p*-value; Ova, ovalbumin; PCA, principal component analysis; pTregs, peripherally induced regulatory T cells; RPKM, reads per kilobase of exon length per million reads; SPF, specific pathogen-free; TCR, T cell receptor; TSS, transcriptional start site

Using ATAC-seq, we identified 40,166 differentially accessible regions (DARs) across all conditions, among which a substantial proportion (26.1 %) was located at transcriptional start sites (TSSs) (Supplementary Fig. S7). Global inspection of the DARs at the TSSs via principal component analysis (PCA) revealed the expected segregation of naive T cells and *de novo* induced pTregs, but we did not identify substantial changes between GF and SPF mouse pTregs (Fig. 4a, left). Regarding differentially expressed genes (DEGs) from the RNA-seq analysis, PCA showed a segregation of naive T cells and *de novo* induced pTregs, with a clear clustering of the SPF and GF replicates. However, microbiota-dependent differences were marginal in comparison to the transcriptional changes instigated upon TCR-based stimulation (Fig. 4a, right). Hierarchical clustering of all DARs at the TSSs (*n*_Genes_ = 5134) (Fig. 4b, left) and all DEGs (*n*_Genes_ = 837) (Fig. 4b, right) further substantiated this finding, revealing a nearly identical transcriptional signature and chromatin landscape between GF and SPF mouse pTregs. In fact, when directly comparing SPF to GF samples, only four DEGs could be identified (Fig. 4c), while no DARs were identified within the TSSs, intragenic or intergenic regions (data not shown). The four DEGs *Slc16a5* (solute carrier family 16 member 5), *Rtp4* (receptor transporting protein 4), *Pfkfb3* (fructose-2,6-biphosphatase 3), and *Zfp275* (zinc finger protein 275) (Fig. 4d) are, to the best of our knowledge, not specifically linked to Treg biology. Thus, in line with the comparable frequency of *de novo* induced pTregs in the mLNs of GF and SPF-housed mice, we found no evidence that the microbiota is able to modulate the transcriptomic landscape and chromatin accessibility of newly generated pTregs in mLNs.

**Fig. 5:**
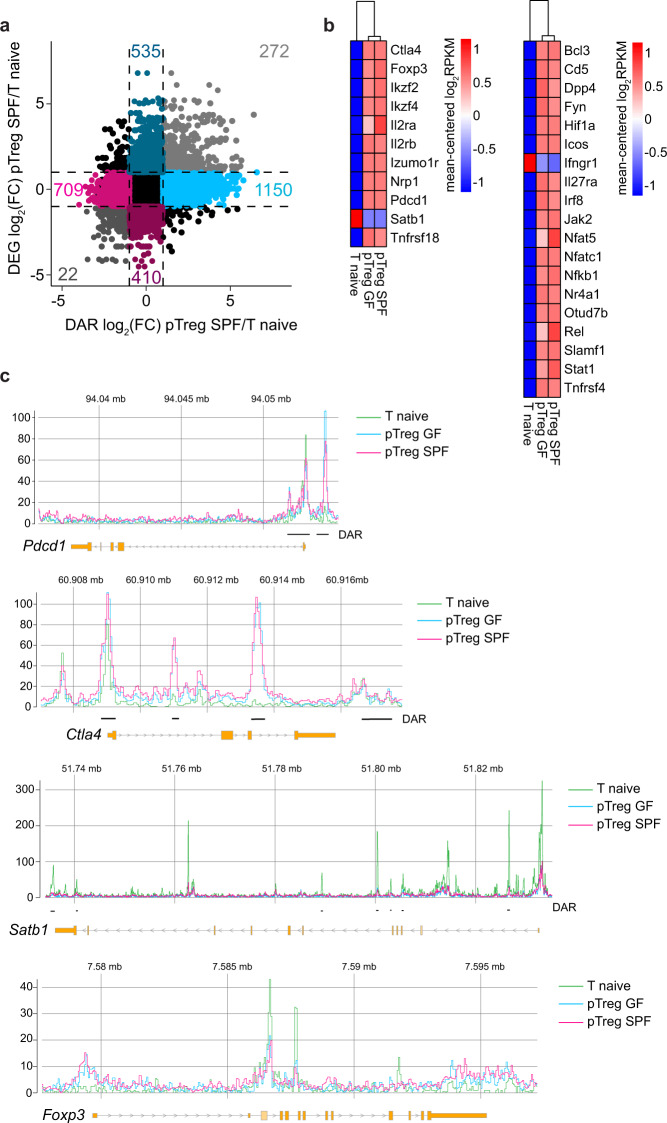
Treg signature genes are upregulated at early stages of pTreg induction. *De novo* induced Foxp3^hCD2+^ pTregs among adoptively transferred CD4^+^OvaTCR^+^ T cells were isolated from mLNs of GF and SPF-housed mice after Ova immunization, and CD62L^+^CD4^+^OvaTCR^+^ naive T cells were freshly isolated from Foxp3^hCD2^xRag2^*−/−*^xDO11.10 mice. RNA-seq and ATAC-seq analyses were performed. DEGs and DARs in the TSS were identified in colonization- (SPF vs. GF) and cell-type-dependent (pTregs vs naive T cells) pairwise comparisons (log_2_(FC) ≥ 1, *q*-value ≤ 0.05). **a** Scatterplot comparing the gene-wise fold change of the chromatin accessibility and transcriptome between SPF pTregs/naive T cells. On the *x*-axis, log_2_(FC) of mean fold change of DARs per gene, and on the *y*-axis, log_2_(FC) of gene expression are plotted. Colored numbers in the scatterplot represent the number of genes in the indicated quadrant. **b** Heatmaps depict signature Treg genes across all experimental groups **(left)** and genes involved in NF-κB, T cell receptor, NFAT and interferon signaling **(right)**. **c** Genome tracks depict mean-replicate values of ATAC-seq FPKM for *Foxp3*, *Clta4*, *Satb1*, and *Pdcd1*. Datasets are group-normalized to maximum peak height, as indicated on the *y*-axis. Highlighted DARs are significant for at least one pairwise comparison. Samples for both ATAC-seq and RNA-seq were pooled from two independent experiments (*n* = 2–4). ATAC, assay for transposase accessible chromatin; DAR, differentially accessible region; DEG, differentially expressed gene; FC, fold change; FPKM, fragments per kilobase of peak per million reads; GF, germ-free; mLNs, mesenteric lymph nodes; Ova, ovalbumin; pTregs, peripherally induced regulatory T cells; RPKM, reads per kilobase of exon length per million reads; SPF, specific pathogen-free; TCR, T cell receptor; TSS, transcriptional start site

Upon comparison of the interplay between chromatin accessibility and transcription among SPF mouse pTregs and naive T cells on a per-gene basis, 1859 genes were differentially accessible comparing SPF mouse pTregs to naive T cells (DARs only), 945 genes were up/downregulated in SPF mouse pTregs compared to those in naive T cells (DEGs only), and 294 genes were both up/downregulated and showed differential accessibility in SPF mouse pTregs compared to naive T cells (DARs&DEGs) (Fig. 5a). To identify functional consequences related to the changes in the chromatin landscape and gene expression in SPF mouse pTregs, we performed a gene ontology (GO) analysis on the three sets of genes (DARs only, DEGs only, and DARs&DEGs). We identified key biological processes related to cell proliferation (GO: 0050798, GO: 0042130, GO: 0030888, GO: 0001787, GO: 0032946) and T cell activation (GO: 0006924, GO: 0031295, GO: 0042104, GO: 0030098) (Supplementary Fig.  S8). Both SPF and GF mouse pTregs upregulated characteristic Treg signature genes when compared to naive T cells and showed diminished expression of *Satb1* (Fig. 5b, left), confirming previously published results.^45,46^ In addition, multiple genes involved in TCR-mediated T cell activation, including NF-κB and interferon signaling, were upregulated in SPF and GF mouse pTregs compared to naive T cells (Fig. 5b, right; Supplementary Fig. S8). In accordance with these gene expression patterns, *Ctla4* and *Pdcd1* displayed significantly more accessible chromatin in both SPF and GF mouse pTregs than in naive T cells, while the accessibility of *Satb1* was diminished (Fig. 5c). For the *Foxp3* locus, surprisingly, aside from a slightly increased accessibility in the promoter region that did not reach significance, none of the open regions within SPF or GF mouse pTregs showed an elevated accessibility when compared to those of naive T cells (Fig. 5c), which was also the case for *Izumo1r* and *Tnfrsf18* (data not shown).

Together, our data indicate that *de novo* induced pTregs activate gene modules downstream of TCR stimulation and proliferate and express Treg signature genes in a comparable manner in mLNs of both GF and SPF-housed mice, further supporting the key finding of the present study that the microbiota is dispensable during the early stages of pTreg induction in mLNs.

## Discussion

Previous work emphasized the role of SCFAs as microbiota-derived metabolic contributors to intestinal Treg homeostasis.^30–32^ However, in the present study, we did not observe enhanced *de novo* pTreg induction within the mLNs upon dietary supplementation with SCFAs. However, it is important to note that the applied SCFA treatment regimen also did not cause increased frequencies or numbers of Foxp3^+^ Tregs within the colonic lamina propria, as previously reported.^30–32^ It is highly unlikely that dietary SCFA supplementation was too short, as it lasted for up to nine weeks throughout gestation, weaning and early adulthood, which is substantially longer than the 3-week treatment reported by Smith et al.^31,32^ In addition, the applied SCFA concentrations (100 mM) were likely sufficient, which were in the range of the concentrations used previously (100–150 mM), although it is important to note that 50 mM individual SCFAs were found to be insufficient to increase Foxp3^+^ Tregs within the colonic lamina propria.^31,32^ Alternatively, the discrepancies between the studies could be indicative of a ceiling effect, where SCFAs produced by the microbiota and diet are already exerting their maximal effect on the intestinal Treg compartment, which cannot be further enhanced by dietary SCFA supplementation. Although mild effects on the induction of CCR9 expression were observed upon acetate treatment, the findings from the present study suggest that mild alterations of SCFA levels do not readily modulate the pTreg-inducing capacity of mLNs.

In contrast to microbiota-derived metabolites, such as SCFAs, microbes are actively prohibited from passing the epithelial barrier.^47^ Despite the effective barrier function of the intestine, distinct microbial communities are readily able to instigate proinflammatory responses.^48,49^ However, in our study, despite the microbiota containing *Prevotellaceae* pathobionts, pTreg induction within mLNs was completely unaffected. As compared to the colonic lamina propria,^47^ mLNs are largely shielded from the microbiota, it is likely that the proinflammatory effects of the transferred dysbiotic microbiota are contained in the lamina propria under steady-state conditions, assuring that the intrinsic tolerogenic properties of mLNs are resistant to fluctuations in microbiota composition. However, the transferred pathobionts could very well affect different sections of the intestine with varying severity, potentially resulting in mild alterations of pTreg induction within individual mLNs along the intestine.^13^

In addition, data from the present study even suggest that the microbiota and microbiota-derived metabolites do not affect the early stages of pTreg induction in mLNs at all, as equally high pTreg induction was observed in the mLNs of GF mice compared to that in the mLNs of age-matched SPF-housed control mice. These results are in accordance with previously published findings that eradication of the microbiota in adult mice using a cocktail of antibiotics did not affect the pTreg-inducing capacity of mLNs.^16^ However, although these data nicely concur with the abovementioned assumption that the mLNs are largely shielded from the microbiota and their metabolites, this finding was still surprising, as we had previously demonstrated that the mLNs from GF mice lose their high Treg-inducing capacity upon transplantation into a skin-draining site, while the mLNs from SPF-housed mice could stably maintain their tolerogenic properties long-lastingly.^16,42^ It is important to note that by systemically applying Ova peptide via i.v. administration, the direct effect of the microbiota on LN-resident cells and LN-intrinsic pTreg induction can be assessed. This mode of antigen application circumvents the more complex antigen sampling in the gut lumen, subsequent processing by DCs, and finally DC migration from the intestinal lamina propria to the draining LN, which are all processes already known to be affected by the microbiota.^13,20,50,51^ Thus, by systemically applying Ova peptide via i.v. administration, any impact of these factors can be ruled out, which further underscores the robustness of the pTreg induction-supportive environment of the mLNs.

Consistent with equal pTreg induction, the transcriptional and epigenetic landscape of pTregs induced in mLNs of GF versus SPF-housed mice were largely identical. Furthermore, *de novo* induced pTregs from the mLNs of both GF and SPF-housed mice upregulated gene modules involved in TCR signaling, NF-κB/Rel and NFAT signaling, key hallmarks of T cell activation, driving proliferation and Treg fate specification.^52,53^ However, a substantial proportion of the Treg signature genes showed increased expression when compared to those in naive T cells but not necessarily higher accessibility for those same genes, which might partly be due to the transient Ova-mediated stimulation in the experimental TCR-transgenic monoclonal DO11.10 system used in the present study.

The DO11.10 system is limited to address the comprehensive entity of polyclonal T cell responses, as antigen availability and TCR stimulation strength are known to influence Treg cell fate choice.^54^ Despite these limitations, TCR transgenic systems are still the most viable method to address early T cell fate choices related to initial priming in the tissue-draining LN. However, to the best of our knowledge, it is presently impossible to use monoclonal TCR systems to assess pTreg induction in the context of chow-derived antigens, which were shown to readily impact Treg frequencies in the small intestinal lamina propria.^9^ Thus, it will be interesting to study the relative impact of TCR specificity, antigen availability and microbial colonization on pTreg induction in mLNs using more physiological polyclonal systems in the future.

Based on the present data, we hypothesize that mLNs likely predispose *de novo* induced pTregs to a state of susceptibility for subsequent tissue-specific modulation, and it is likely that the microbiota largely contributes to the acquisition of the unique phenotype, subset composition and transcriptome observed among colonic Tregs.^43,44,55^ As Tregs display a large diversity across a multitude of tissues,^44,56–59^ the importance of an initial priming of such tissue-specific phenotypes in secondary lymphoid organs is best exemplified by visceral adipose tissue (VAT) Tregs, which undergo a terminal differentiation step within the VAT after an initial priming in the spleen.^60^ It is thus reasonable to conclude that *de novo* generation of gut-tropic Tregs in mLNs follows the genesis of VAT Tregs in a similar fashion, with the first priming step taking place in mLNs and subsequent terminal differentiation within the intestinal lamina propria.

In conclusion, the present study underpins gut-draining mLNs as key hubs for the initial antigen-specific priming of *de novo* induced pTregs, a process independent of microbial colonization, composition and metabolites.

## Materials and methods

### Mice

Foxp3^hCD2^xRag2^*−/−*^xDO11.10 (BALB/c), Foxp3^hCD2^xCD90.1 (BALB/c), Nlrp6^*−/−*^ (C57BL/6), CD45.1 (BALB/c), CD90.1 (BALB/c), and BALB/c mice were bred and housed under SPF conditions in ventilated, isolated cages at the Helmholtz Centre for Infection Research (Braunschweig, Germany). GF mice (BALB/c) were established by cesarean section (Hannover Medical School, Hannover, Germany) and kept in static isolators at the Helmholtz Centre for Infection Research. In all experiments, mice were sex- and age-matched and fed water and an Ova-free diet ad libitum. Mice were housed and handled in accordance with good animal practice as defined by Federation of European Laboratory Animal Science Associations and the national animal welfare body GV-SOLAS (Society for Laboratory Animal Science). Animal experiments were conducted in compliance with German animal protection law (TierSchG BGBl. I S. 1105; 25.05.1998) and approved by the Lower Saxony Committee on the Ethics of Animal Experiments, as well as the responsible state office (Lower Saxony State Office of Consumer Protection and Food Safety) under permit number 33.9-42502-04-12/1012.

### Cell isolation

Cells were isolated from skin-draining pLNs (inguinal and axial), gut-draining mLNs and the spleen. Single-cell suspensions were prepared by meshing the organs through a 100 µm filter. For spleen samples, erythrocytes were lysed for 3 min with erythrolysis buffer (0.01 M potassium bicarbonate (Merck), 0.155 M ammonium chloride (Roth), 0.1 mM EDTA (Roth), pH 7.5). For cell isolation from the intestine, the colon was cut longitudinally, emptied and washed two times for 15 min at 37 °C in PBS containing 0.2% bovine serum albumin (BSA, Sigma-Aldrich) and 5 mM EDTA (Sigma-Aldrich) to remove the remaining mucus. Colon samples were cut into small pieces and disintegrated in digestion buffer (RPMI-1640 (Gibco) containing 1 mg/ml Collagenase D (Roche), 100 µg/ml DNase I (Roche), and 0.1 U/ml Dispase (Roche)) for 60 min at 37 °C on a rotating shaker. Colonic cells were subsequently washed with PBS containing 0.2% BSA and filtered through a 100 µm sieve before the lymphocyte fraction was separated and harvested using a 40%/80% Percoll (GE Healthcare) gradient in RPMI-1640 (20 min at 780 × *g* at RT without acceleration/deceleration). Cells from all samples were washed and resuspended in PBS containing 0.2% BSA prior to antibody staining for flow cytometric analysis.

### Antibodies, flow cytometry and cell sorting

The following fluorochrome-conjugated antibodies were purchased from BD, Biolegend and eBioscience: anti-(human)-CD2 (RPA-2.10), anti-CD3 (500A2), anti-CD4 (RM4-5), anti-CD8α (53-6.7), anti-CD11c (N418), anti-Ova-TCR (KJ1.26), anti-CD62L (MEL14), anti-CD103 (2E7), anti-CCR9 (CW-1.2), anti-MHCII (M5/114.15.2), anti-Foxp3 (FJK-16S), anti-Helios (22F6), and anti-RORγt (B2D). Flow cytometric analysis was performed as described recently.^61^ In brief, single-cell suspensions were washed with PBS before viability staining was carried out using a LIVE/DEAD™ Fixable Blue Dead Cell Stain Kit (Invitrogen) according to the manufacturer’s instructions. To prevent nonspecific antibody binding, cell suspensions were incubated with 10 µg/ml Chrom Pure rat IgG (Jackson ImmunoResearch Laboratories) and 10 µg/ml anti-mouse CD16/CD32 antibody (BioXcell). For surface staining, cells were washed and then incubated with the corresponding antibodies dissolved in PBS containing 0.2% BSA for 15 min on ice. Intracellular staining was carried out with a Foxp3/Transcription Factor Staining Set (eBioscience) according to the manufacturer’s instructions. Samples were resuspended in PBS containing 0.2% BSA before labeled cells were acquired on an LSRII or LSR Fortessa (BD Biosciences) flow cytometer with Diva software (BD Bioscience). Acquired data were analyzed utilizing FlowJo software (Tree Star). Cell sorting for RNA-seq and ATAC-seq analyses was performed at the cell sorting facility of the Helmholtz Centre for Infection Research on a FACS Aria IIu (BD Bioscience) or FACS Aria II SORP (BD Bioscience). For cell counts, an aliquot of cells was stained with propidium iodide (Sigma) according to the manufacturer´s instructions to label dead cells before samples were analyzed on an Accuri flow cytometer (BD Bioscience).

### T cell isolation for adoptive transfer and Ova immunization

Single-cell suspensions from the spleen and LNs of Foxp3^hCD2^xRag2^*−/−*^xDO11.10 mice were generated as described above, and cells were labeled with CPDviolet (Invitrogen) to track cell proliferation before being adoptively transferred. Approximately 3–10 million cells in 100 µl of PBS were administered via i.v. injection per recipient mouse. Starting one day after adoptive transfer, 20 µg of Ova_323-339_ peptide was administered via i.v. injection on two consecutive days. On day 3 after antigen application, recipient mice were sacrificed, and cells were isolated from the indicated endogenous LNs for flow cytometric analysis or cell sorting. The model system of systemically applying Ova peptide via i.v. injection is well established in our laboratory^16,42,62^ and permits the study of antigen-specific T cell differentiation across all LNs, as it circumvents prior antigen uptake and processing steps. By adoptively transferring 3–10 million cells, a sufficient number of transferred T cells can be reisolated subsequent to Ova peptide application,^13,20,63^ which is a prerequisite to study early T cell differentiation events in particular.

### SCFA supplementation

Propionate, butyrate and acetate (all Sigma-Aldrich) were added at 100 mM to the drinking water, and supplemented water flasks were changed two times per week. SCFA supplementation was initiated in pregnant BALB/c mice at E7 during gestation and continued throughout birth and the weaning period until the offspring reached six to seven weeks of age.

### Transfer of colitogenic microbiota

Seven- to twelve-week-old female *Nlrp6*^*−/−*^ (C57BL/6) mice were cohoused with 5- to 6-week-old female SPF mice (BALB/c) for 4 weeks. As a control, age-matched female wild-type C57BL/6 mice were cohoused with female SPF mice (BALB/c). Subsequent to cohousing, the female BALB/c mice of both experimental groups were mated to obtain offspring of six to seven weeks of age to assess *de novo* pTreg induction.

### 16S rDNA-seq

Fecal pellets were stored at −80 °C until they were processed as previously described.^64^ In brief, fecal samples were suspended in 500 µl of extraction buffer (200 mM Tris, 20 mM EDTA, 200 mM sodium chloride (pH 8.0)), 200 µl of 20% sodium dodecyl sulfate, 500 µl of phenol/chloroform/isoamyl alcohol (14/24/1), and 100 µl of zirconia/silica beads (0.1 mm diameter) for subsequent DNA extraction. Samples were homogenized twice for 2 min per cycle with a bead beater (BioSpec). Crude DNA extracts were resuspended in TE buffer containing 100 µg/ml RNAse I and subsequently column purified to remove PCR contaminants. Following DNA extraction, the V4 region (F515/R806) of the 16S rRNA gene was amplified as previously described.^65^ In short, for amplicon sequencing, 25 ng of DNA was utilized per 30 µl reaction in a 25-cycle PCR (10 s at 98 °C, 20 s at 55 °C, and 20 s at 72 °C), following initial denaturation for 30 s at 98 °C. PCR amplicons were pooled and sequenced on the Illumina MiSeq using 250 bp paired-end reads. The obtained reads were assembled, quality controlled and clustered with the Usearch 8.1 software package. The reads were merged utilizing –*fastq_mergepairs* with *–fastq_maxdiffs 30* before quality control filtering was conducted with *fastq_filter -fastq_maxee 1* and a minimum read length of 200 bp. The UPARSE algorithm was used to determine operational taxonomic unit (OTU) clusters and representative sequences. Taxonomy assignment was carried out by referring to the Silva database v128 and the RDP Classifier using a bootstrap confidence cut off of 80%. Only phylotypes exhibiting a cumulative abundance of at least 0.1% and a sequence length >200 bp were considered for follow-up analysis. The OTU absolute abundance table and mapping file were further processed for statistical analysis and data visualization in R with the *phyloseq*, *ggplot2* and *pheatmap* packages.

### RNA-seq

Foxp3^hCD2+^CD4^+^OvaTCR^+^ Tregs and CD62L^+^CD4^+^OvaTCR^+^ naive T cells were directly FACS sorted into RLT^+^ lysis buffer (Qiagen) containing 10 µl/ml β-mercaptoethanol (Roth). Total RNA was extracted with an RNeasy Plus Micro Kit (Qiagen) according to the manufacturer’s instructions. cDNA conversion was carried out using a SMART-Seq v4 Ultra Low Input RNA Kit (Takara Clontech Laboratories) according to the manufacturer’s instructions, followed by PCR product purification with Agencourt AMPure XP magnetic beads (Beckman Coulter). Libraries were prepared with a Nextera XT DNA Library Prep Kit (Illumina) employing enzymatic tagmentation and simultaneous adapter ligation before subsequent indexing and fragment amplification. PCR product clean-up and size selection were performed with Agencourt AMPure XP magnetic beads. RNA and cDNA quality and content, as well as the correct size of cDNA library fragments, were verified using Agilent Technologies 2100 Bioanalyzer profiles and Qubit measurements after each step. Libraries were pooled and sequenced at the genome analytics facility of the Helmholtz Centre for Infection Research on an Illumina HiSeq2500 using 50 bp single-end reads. The read quality of sequenced libraries was evaluated with *FastQC*. Sequencing reads were aligned to the reference mouse genome assembly GRCm38 using STAR, and reads aligned to annotated genes were quantified with *htseq-count*.^66^ The calculated read counts were further processed with *DESeq2* for quantification of differential gene expression.^67^ Raw read counts were converted to reads per kilobase of exon length per million mapped reads (RPKM) values. Only genes with an annotated *Gene Symbol* are depicted in plots. GO analysis was performed using *topGO*.^68^ Graphics were generated in R using *pheatmap* and *ggplot2*. The Gene Expression Omnibus (GEO) accession number for the RNA-seq data reported in this paper is GSE149851.

### ATAC-seq

Foxp3^hCD2+^CD4^+^OvaTCR^+^ Tregs and CD62L^+^CD4^+^OvaTCR^+^ naive T cells were FACS sorted into PBS containing 0.2% BSA. Cells were washed once with PBS before DNA transposition was performed with a Nextera DNA Library Prep Kit (Illumina). Per sample, 25 µl of TD, 2.5 µl of TDE1, and 22 µl of nuclease-free water were combined and placed at 37 °C for 3 min before 0.5 µl of 1% Digitonin (Promega) was added to the master mix.^69^ Samples were resuspended in the transposition reaction mix and incubated for 30 min at 37 °C at 300 rpm. After transposition, DNA was purified with a MinElute PCR Purification Kit (Qiagen) according to the manufacturer’s instructions and eluted in 50 µl of nuclease-free water. Transposed DNA fragments were preamplified using 10 μl of transposed DNA, 10 μl of nuclease-free water, 2.5 μl of 25 μM custom Nextera PCR primer 1, 2.5 μl of 25 μM custom Nextera PCR primer 2, and 25 μl of NEBNext High-Fidelity 2× PCR Master Mix (New England BioLabs) per reaction and amplified via a 6-cycle PCR program (1 cycle of 72 °C for 5 min and 98 °C for 30 s; 5 cycles of 98 °C for 10 s, 63 °C for 30 s, and 72 °C for 1 min). The forward primer was identical for all samples (5′-AATGATCGGCGACCACCGAGATCTACACTCGTCGGCAGCGTCAGATGTG-3′), whereas the reverse primer contained distinct barcodes (example underlined) used for demultiplexing (5′-CAAGCAGAAGACGGCATACGAGAT*TCGCCTTA*GTCTCGTGGGCTCGGAGATGT-3′).^70^ The appropriate number of further amplification cycles was determined by qPCR using 5 µl of the preamplified product. Final amplification was carried out with 45 μl of previously PCR-amplified DNA, 39.7 μl of nuclease free water, 2.25 μl of 25 μM customized Nextera PCR primer 1, 2.25 μl of 25 μM customized Nextera PCR Primer 2, 0.81 μl of 100× SYBR Green I, and 45 μl of NEBNext High-Fidelity 2x PCR Master Mix with the PCR program of 1 cycle of 98 °C for 30 s and 8–10 cycles (depending on qPCR results) of 98 °C for 10 s, 63 °C for 30 s, and 72 °C for 1 min. PCR purification was carried out using a MinElute PCR Purification Kit. Finally, size selection was performed with SPRIselect beads (Beckmann-Coulter) with 1.2× for left-side and 0.55× for right-side selection according to the manufacturer’s instructions. DNA quality, content and fragment size were assessed with Agilent Technologies 2100 Bioanalyzer profiles and Qubit measurements. Sequencing of libraries was carried out at the genome analytics facility of the Helmholtz Centre for Infection Research on an Illumina NOVASeq6000 using 50 bp single-end reads, and the quality of sequenced libraries was verified with *FastQC*. Sequencing reads were mapped to the mouse genome (mm10) using *STAR* (version 2.5.3a)^71^ with the parameters *--runMode alignReads --outSAMtype BAM SortedByCoordinate --outFilterMultimapNmax 1*. Duplicates were removed using *Picard MarkDuplicates* (https://broadinstitute.github.io/picard/). Peaks were called on deduplicated bam files using *macs2 callpeak* with the parameters *--broad -g mm -q 0.05* for each replicate.^72^ Heatmaps of fragment distribution around the TSS were computed using *computeMatrix* with the *reference-point -a 3000 -b 3000* and plotted using *plotHeatmap*. The regions identified via *macs2* were merged across all replicates into one set of regions by combining peaks overlapping with at least one base pair and removing peaks that overlapped with blacklisted regions.^73^ Differential accessibility of raw ATAC-seq counts for each region/peak across all replicates of all samples was normalized across replicates with size factors computed with *DESeq2* (version 1.22).^67^ Pairwise comparisons were performed with *DESeq2*, and DARs were called with an FDR adjusted p-value of less than 0.05 and a fold change (FC) of at least 2. Genomic features were identified via *getAnnotation* from *ChIPpeakANNO*.^74^ The cumulative FC of all DARs for one respective gene is represented as the mean of all the FCs of all respective DARs. Graphics were generated in R using *pheatmap* and *ggplot2*. The GEO accession number for the ATAC-seq data reported in this paper is GSE150566.

### Statistical analysis

Prism software (GraphPad) was utilized for statistical analysis of flow cytometry-based data. For all figures, if not stated otherwise, each data point represents a single mouse. When unmatched groups were compared, a two-tailed Mann–Whitney test was applied. Data are presented as the mean ± SD, and *p* values below a threshold of 0.05 were considered significant; *p < 0.05; **p < 0.01; ***p < 0.001; ****p < 0.0001; ns = not significant.

## Supplementary information

Supplementary Figure 1

Supplementary Figure 2

Supplementary Figure 3

Supplementary Figure 4

Supplementary Figure 5

Supplementary Figure 6

Supplementary Figure 7

Supplementary Figure 8
