# Supplementary figures and images for: The microbiota is dispensable for the early stages of peripheral regulatory T cell induction within mesenteric lymph nodes
Source: Cell Mol Immunol. 2021 Mar 24;18(5):1211–21. doi: 10.1038/s41423-021-00647-2 (PMC8093251; doi:10.1038/s41423-021-00647-2)

# Supplementary Figure 1

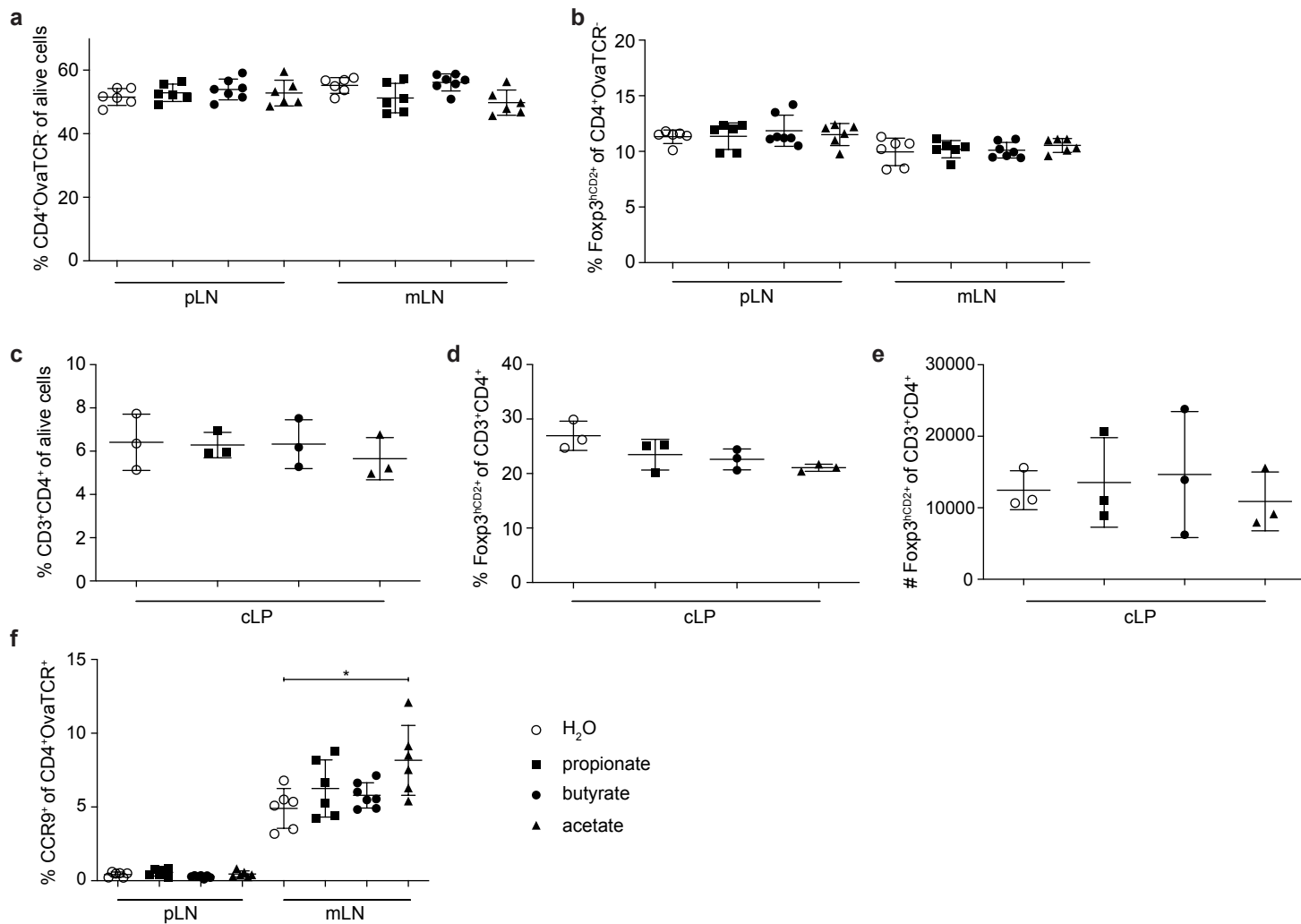

Supplement: Supplementary file 1 — Supplementary Figure 1 [file 41423_2021_647_MOESM1_ESM.pdf]

**Supplementary Figure 2**

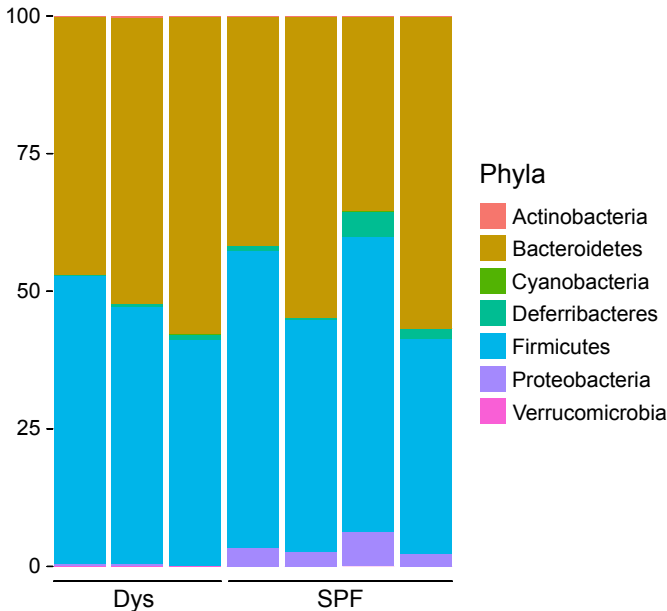

Supplement: Supplementary file 2 — Supplementary Figure 2 [file 41423_2021_647_MOESM2_ESM.pdf]

**Supplementary Figure 3**

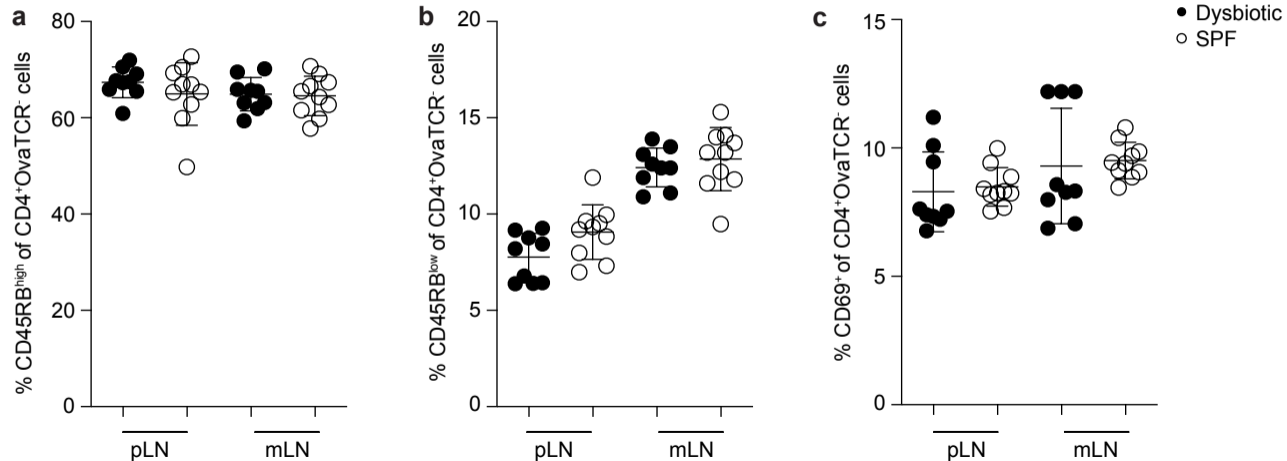

Supplement: Supplementary file 3 — Supplementary Figure 3 [file 41423_2021_647_MOESM3_ESM.pdf]

**Supplementary Figure 4**

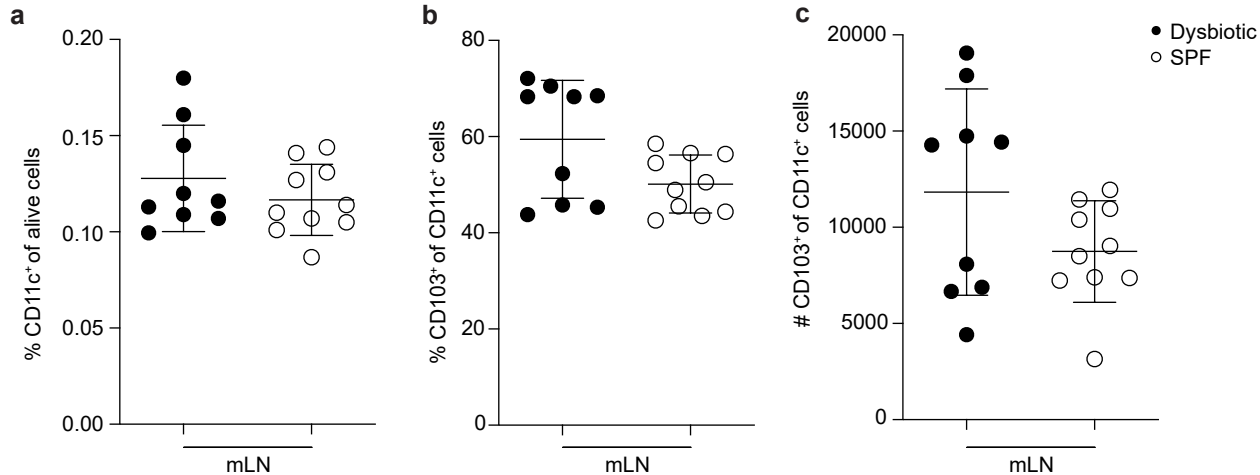

Supplement: Supplementary file 4 — Supplementary Figure 4 [file 41423_2021_647_MOESM4_ESM.pdf]

## Supplementary Figure 5

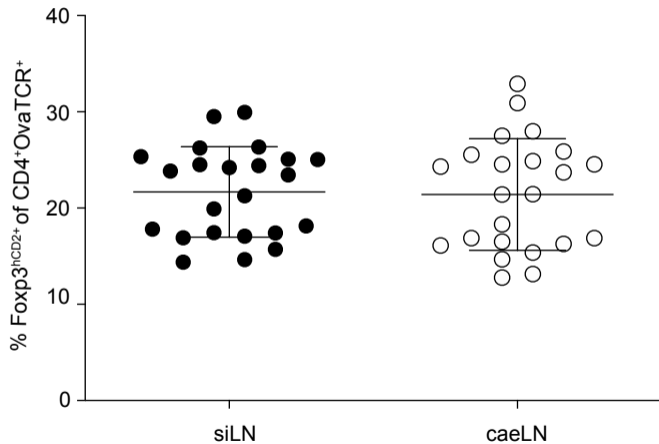

Supplement: Supplementary file 5 — Supplementary Figure 5 [file 41423_2021_647_MOESM5_ESM.pdf]

# Supplementary Figure 6

**a**

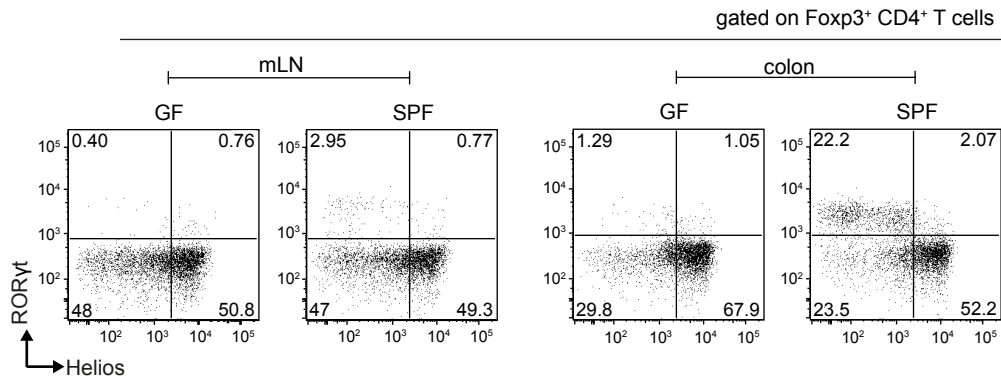

**b**

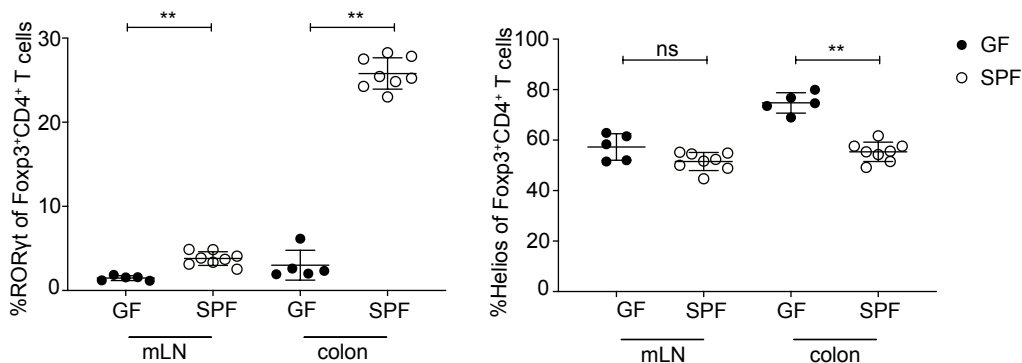

Supplement: Supplementary file 6 — Supplementary Figure 6 [file 41423_2021_647_MOESM6_ESM.pdf]

# Supplementary Figure 7

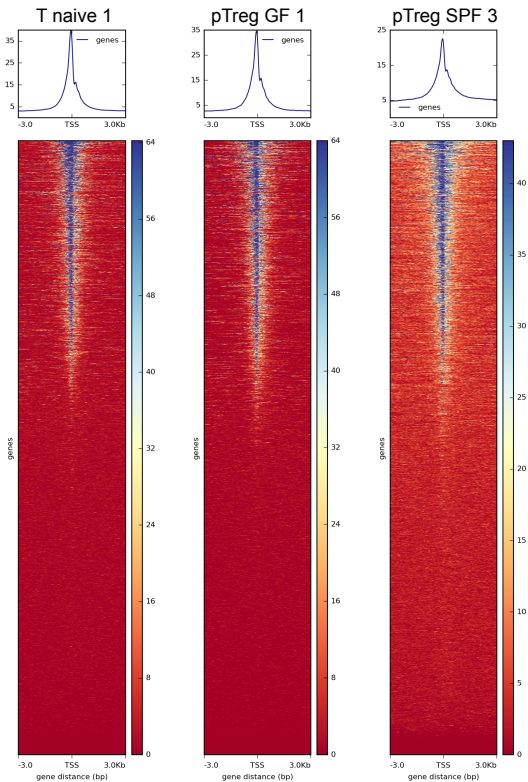

Supplement: Supplementary file 7 — Supplementary Figure 7 [file 41423_2021_647_MOESM7_ESM.pdf]

Supplementary Figure 8

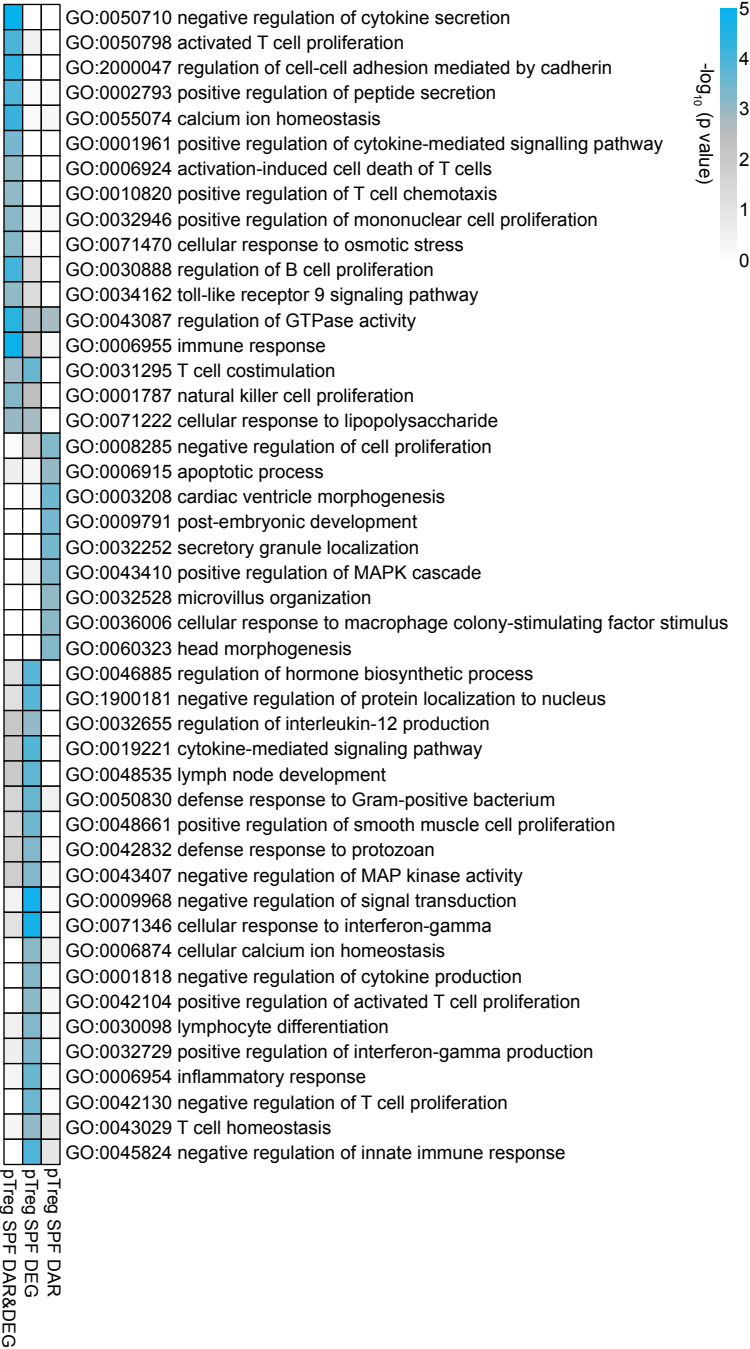

Supplement: Supplementary file 8 — Supplementary Figure 8 [file 41423_2021_647_MOESM8_ESM.pdf]
